# Necessity for higher teicoplanin doses in older adults: a multicenter prospective observational study in China

**DOI:** 10.1186/s12877-024-05091-1

**Published:** 2024-06-04

**Authors:** Tingting Liu, Jionghe Wu, Peng Na, Xia Wu, Yaping Yuan, Chao Wang, Xuewei Ma, Lin Qi, Xiaomin Chen, Weiqiao Rao, Zhimei Duan, Xiangqun Fang, Lixin Xie, Hongxia Li

**Affiliations:** 1 Department of Pulmonary and Critical Care Medicine, The Second Medical Center, National Clinical Research Center for Geriatric Diseases, Chinese PLA General Hospital, Beijing, 100853 China; 2 College of Pulmonary & Critical Care Medicine, Chinese PLA General Hospital, Beijing, 100853 China; 3 Department of Pulmonary and Critical Care Medicine, The Fourth Medical Center, Chinese PLA General Hospital, Beijing, 100853 China; 4 Department of Pulmonary and Critical Care Medicine, The First Medical Center, Chinese PLA General Hospital, Beijing, 100853 China; 5 Chinese PLA Medical School, Beijing, 100853 China; 6 BGI Genomics Co., Ltd, Shenzhen, 518083 China

**Keywords:** Teicoplanin, Therapeutic drug monitoring, Dose regimen, Toxicity, Older adults

## Abstract

**Background:**

Many older adult patients receive low-dose teicoplanin with varied regimens, leading to a lack of clarity on its optimal regimens and toxicity profiles in China. This study aimed to clarify these aspects by analyzing teicoplanin treatment concentrations and toxicities.

**Methods:**

We included older adult patients administered teicoplanin at four tertiary hospitals in Beijing from June 2021 to July 2023, targeting a trough concentration (C_min_) ≥ 10 mg/L. Teicoplanin concentrations and toxicities were monitored dynamically.

**Results:**

From 204 patients, we obtained 632 teicoplanin concentrations. Most patients (83.3%) received low-dose regimens. Suboptimal concentrations were found in 66.4% of patients within 7 days of treatment and 17.0% after 15 days. C_min_ gradually increased with treatment duration and was influenced initially by creatinine and by both body weight and creatinine from days 8 to 14. The target concentration was achieved in 53.1%, 33.9%, 15.6%, and 5.5% of patients at 3, ≤ 7, 8–14, and ≥ 15 days after withdrawal, respectively. Slow elimination was associated with average C_min_ and eGFR. Nephrotoxicity, hepatotoxicity, and thrombocytopenia occurred in 12.5%, 4.1%, and 31.5% of patients, respectively, without significant differences between concentrations.

**Conclusions:**

Most older adult patients were underdosed, indicating a need for dose adjustment. Given the varied risk factors for suboptimal concentrations in different treatment stages, a one-size-fits-all regimen was ineffective. We recommend an initial dose of 400 mg at 12-h intervals for the first three days, with subsequent doses from days 4 to 14 adjusted based on creatinine and body weight; after day 14, a maintenance dose of 200 mg daily is advised.

**Trial registration:**

ChiCTR2100046811; 28/05/2021.

## Background

*Staphylococcus aureus* accounts for approximately 15% of infections within intensive care units worldwide; methicillin-resistant *S. aureus* (MRSA) is responsible for about a third of these, often leading to significantly high mortality rates [1]. In older adults, the convergence of factors such as multiple comorbidities, extensive polypharmacy, diminished immune response from aging (immunosenescence), and increased frailty amplifies the risk of MRSA infections [2]. Given numerous studies indicating that the effectiveness of teicoplanin rivals that of vancomycin with a notably lower adverse reaction rate, its use has become widespread for treating these infections [3–5].

The efficacy of teicoplanin is closely linked to its pharmacokinetic/pharmacodynamic properties, with the ratio of the area under the concentration–time curve to the minimum inhibitory concentration being a key indicator [6, 7]. The trough concentration (C_min_) has been identified as a valuable alternative metric because of its strong linear correlation with the area under the concentration–time curve [8, 9]. Clinical evidence suggests that a C_min_ ranging from 10 to 20 mg/L is associated with positive outcomes when treating uncomplicated infections, whereas more severe infections, such as endocarditis and osteomyelitis caused by staphylococci, may require higher concentrations (20 to 30 mg/L) [6, 7]. The summary of product characteristics for teicoplanin suggests a loading dose of 400 mg (6 mg/kg) administered every 12 h for the initial three doses, followed by a 400 mg daily maintenance dose for most Gram-positive bacterial infections; for severe infections, it recommends increasing the loading and maintenance doses as well as the target C_min_, although recommendations vary internationally [10–18].

As a hydrophilic, renally cleared, highly protein-bound antibiotic, teicoplanin use is challenging in older adults, who often have conditions such as sepsis, renal impairment, and hypoalbuminemia [2] that make them prone to drug pharmacokinetic variability. Despite recent updates in guidelines and expert consensus in China advocating for higher doses to be used in older adults, real-world practices tend to have lower dosing regimens [19], largely because of concerns surrounding nephrotoxicity. However, these lower dosing regimens are not consistent.

This study aimed to bridge the knowledge gap regarding the optimal dosing regimen for teicoplanin in older adults, particularly those over 90 years of age. By examining current dosing practices, serum concentration profiles during treatment and after teicoplanin withdrawal, and associated drug-induced toxicities, we sought to delineate a regimen that maximizes efficacy while minimizing adverse effects in this vulnerable population.

## Methods

### Setting

This prospective, multicenter, open-label observational study was conducted from June 2021 to July 2023 at four tertiary care centers affiliated with the Chinese PLA General Hospital in Beijing, China. The study adhered to the principles of the Declaration of Helsinki and was approved by the hospital's Ethics Committee. Written informed consent was obtained from all participants or their legal guardians.

### Study population

The inclusion criteria were age ≥ 60 years, receipt of teicoplanin, and suspected or confirmed Gram-positive infection. The exclusion criteria were a lack of informed consent, treatment duration ≤ 5 days, receipt of renal replacement therapy, previous enrollment in the study within the past year, and known hypersensitivity to teicoplanin.

### Data collection

Basic information including sex, age, underlying diseases (chronic obstructive pulmonary disease, respiratory failure, hypertension, coronary heart disease, diabetes, chronic kidney dysfunction, or malignant tumor), infection site, duration of teicoplanin therapy, laboratory findings, estimated glomerular filtration rate (eGFR), Sequential Organ Failure Assessment score, receipt of antibiotics, and prognosis was collected for each subject. eGFR was estimated by formula of CKD-EPI.

### Dose regimens

Teicoplanin (Targocid, Sanofi, Dublin, Ireland) was administered intravenously for 30 min. The prescribed dose regimens were at the discretion of treating physicians, and the recommended regimens were not always followed.

### Blood sampling, measurement, and therapeutic drug monitoring (TDM)

Blood samples (5 mL) were collected from the elbow into ethylenediaminetetraacetic acid-containing Vacutainers® (Becton Dickinson, Milan, Italy) in the morning before teicoplanin administration and after drug withdrawal. Samples were promptly refrigerated and centrifuged at 2500 × g for 10 min before 2 mL of supernatant was preserved at -20 °C for subsequent analysis. Teicoplanin concentrations were determined using liquid chromatography-tandem mass spectrometry [20–22]. The linear range of the method was 1.0–100.0 mg/L, and the lower limit of quantification was 1.0 mg/L. The relative standard deviation of intra- and inter-batch precision was ≤ 10%.

**Fig. 1:**
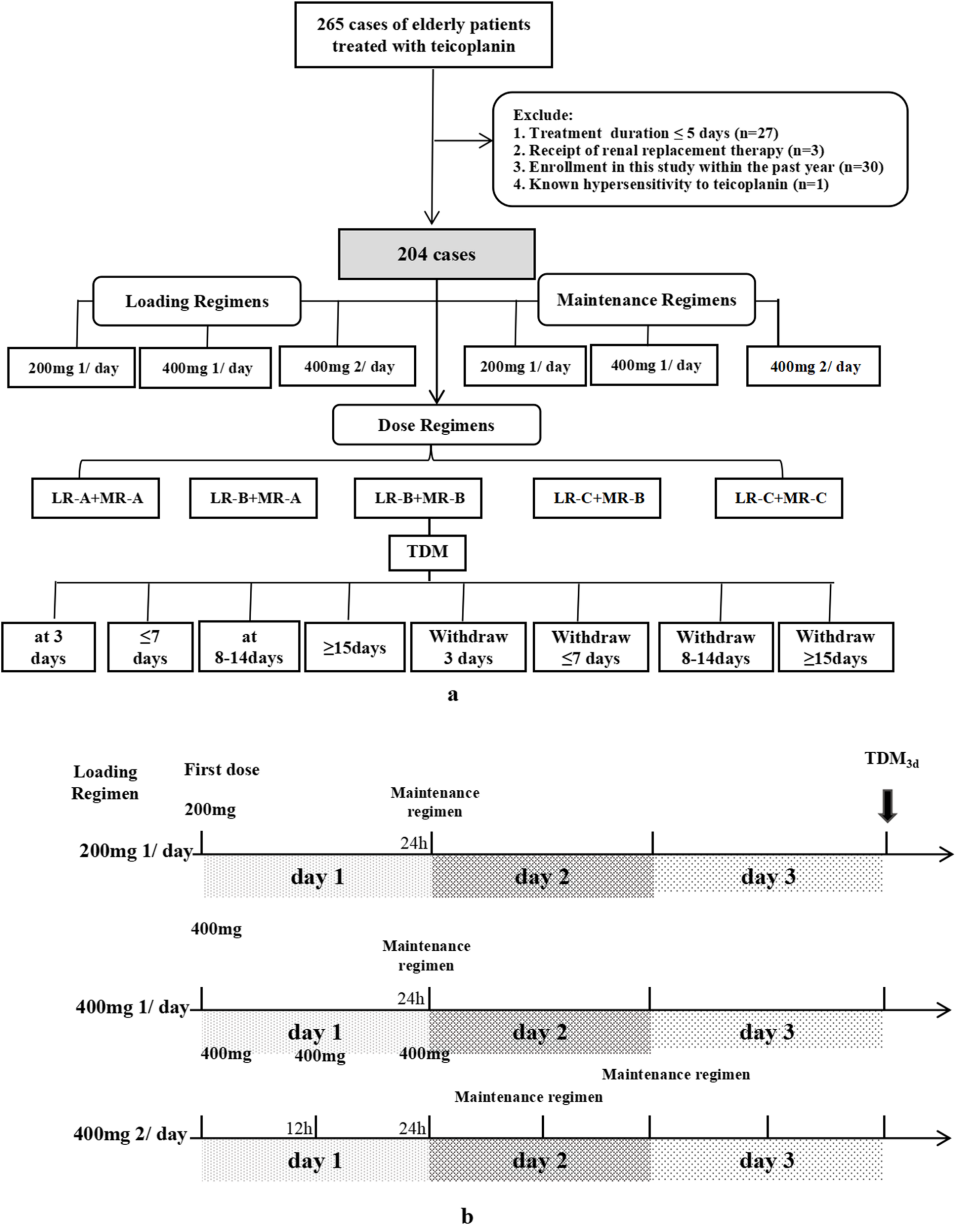
**a** Flow chart of patient enrollment and study design; **b** Loading Regimens and Maintenance Regimens. *TDM, therapeutic drug monitoring*

Teicoplanin concentrations were dynamically monitored. Concentrations at 3, ≤ 7, 8–14, and ≥ 15 days after the first dose and within 2 h of the next scheduled dose were recorded as TDM_3d_, TDM_≤7d_, TDM_8-14d_, and TDM_≥15d_, respectively. Concentrations at 3, ≤ 7, 8–14, and ≥ 15 days after the last teicoplanin dose (withdrawal) were recorded as TDM_w3d_, TDM_w≤7d_, TDM_w8-14d_, and TDM_w≥15d_. respectively (Fig. 1a). The C_min_ target was ≥ 10 mg/L [6, 7, 23]; a concentration < 10 mg/L was considered suboptimal. Average TDM (TDM_a_) was defined as the mean C_min_ after 3 days of treatment.

### Adverse events

Patients with renal impairment at baseline were excluded. Nephrotoxicity was defined as acute renal impairment indicated by a serum creatinine increase of > 50% from baseline [24].

Patients with abnormal liver function at baseline were excluded. Hepatotoxicity was defined as an increase in the alanine aminotransferase or aspartate aminotransferase concentration to more than three times the upper limit of the institution’s normal reference ranges [10, 17].

Patients with platelet counts < 100 × 10^9^/L at baseline were excluded. Thrombocytopenia was defined as a decrease in the platelet count of > 30% from baseline [25].

### Statistical analysis

Statistical analysis was performed using IBM SPSS Statistics 23.0 (IBM, Armonk, NY, USA). The normality of continuous variables was examined using the Kolmogorov–Smirnov test. Quantitative data with a normal distribution were expressed as mean and standard deviation and analyzed by *t*-tests. Quantitative data with a non-normal distribution were presented as median and interquartile range and assessed by the Mann–Whitney U test. Numerical data were compared using χ^2^ or Fisher’s exact probability tests. Correlations between factors were determined by Spearman’s correlation analysis. After the exclusion of collinear factors, those significant at *P* < 0.1 in univariate analysis or considered clinically relevant were included in multivariate analysis. Multivariate logistic regression analysis was used to identify factors leading to suboptimal teicoplanin exposure and slow metabolism. *P* < 0.05 was considered significant.

## Results

### Demographic and clinical characteristics of the included patients

In total, 632 teicoplanin concentrations were collected from 204 patients (Fig. 1a). A summary of the demographic and clinical characteristics of the included patients is provided in Table 1. Patients were 89.3 ± 11.3 years old, and 137 (67.1%) were > 90. The loading regimens (LRs) were divided into LR-A (200 mg once daily), LR-B (400 mg once daily), and LR-C (400 mg at 12-h intervals, at least three doses). The maintenance regimens (MRs) were divided into MR-A (200 mg once daily), MR-B (400 mg once daily), and MR-C (400 mg twice daily). The median dose of the LRs was 6.13 ± 3.55 mg/kg, while that of the MRs was 4.25 ± 2.76 mg/kg.

**Table 1:** Clinical characteristics and laboratory findings of 204 older adult patients

Characteristics		All patients (*n* = 204)
Concentrations, n		632
Age, years, x±s		89.3 ± 11.3
Gender, male, N (%)		187(91.7)
Weight, kg, median (IQR)		65[57,62]
Body mass index, kg/m^2^, median (IQR)		23[20, 26]
Loading Regimens, N (%)		
A	200mg 1/day	83(40.7)
B	400mg 1/day	87(42.6)
C	400mg 2/day	34(16.7)
Maintenance Regimens, N (%)		
A	200mg 1/day	163(79.9)
B	400mg 1/day	27(13.2)
C	400mg 2/day	14(6.9)
Dose Regimens, N (%)		
Loading Regimen A + Maintenance Regimen A		83(40.7)
Loading Regimen B + Maintenance Regimen A		80(39.2)
Loading Regimen B + Maintenance Regimen B		7(3.4)
Loading Regimen C + Maintenance Regimen B		20(9.8)
Loading Regimen C + Maintenance Regimen C		14(6.9)
Loading Regimen, mg/kg		6.13 ± 3.55
Maintenance Regimen, mg/kg		4.25 ± 2.76
Duration, days, median (IQR)		12[7, 17]
Underlying disease, N (%)		
COPD		29(14.2)
Respiratory failure		70(34.3)
Non-invasive ventilation		40(19.6)
Invasive ventilation		30(14.7)
Hypertension		136(66.7)
Coronary Heart Disease		3(1.5)
Stable angina pectoris		113(55.4)
Acute myocardial infarction		3(1.5)
Old myocardial infarction		2(1.0)
Diabetes		67(32.8)
CKD		137(67.2)
Chronic liver disease		16(7.8)
Neurological disease		76(37.3)
Malignant tumor		90(44.1)
Infection sites, N (%)		
Pulmonary infection		172(84.3)
Others		32(15.7)
Laboratory findings		
Albumin, g/L, x ± s		39 ± 14
Creatinine, μmol/L, median (IQR)		83[60,113]
eGFR,ml/min/1.73m^2^, median (IQR)		78[53,104]
Bilirubin, μmol/L,median (IQR)		13.7[8.2,22]
ALT, U/L, median (IQR)		15.9[10.0,25.0]
eGFR < 60ml/min/1.73m^2^, N (%)		92(45.1)
Vasoactive agent, N (%)		44(21.6)
SOFA, median (IQR)		7[4, 10]
30-day mortality, N (%)		40(19.6)
Combination of antibiotics, N (%)		
Carbapenems		113(55.4)
Cephalosporin		70(34.4)
Antifungal drug		31(15.2)

*TDM* therapeutic drug monitoring, *COPD* chronic obstructive pulmonary disease, *CKD* chronic kidney disease, *ALT* Alanine aminotransferase, *AKI* Acute kidney injury, *eGFR* estimated glomerular filtration rate (CKD-EPI), *SOFA* Sequential organ failure assessment

Five dose regimens were identified: LR-A + MR-A, LR-B + MR-A, LR-B + MR-B, LR-C + MR-B, and LR-C + MR-C, given to 83 (40.7%), 80 (39.2%), 7 (3.4%), 20 (9.8%), and 14 (6.9%) patients, respectively (Table 1, Fig. 1a-b).

### Dynamic monitoring of teicoplanin concentrations

#### During treatment

**Fig. 2:**
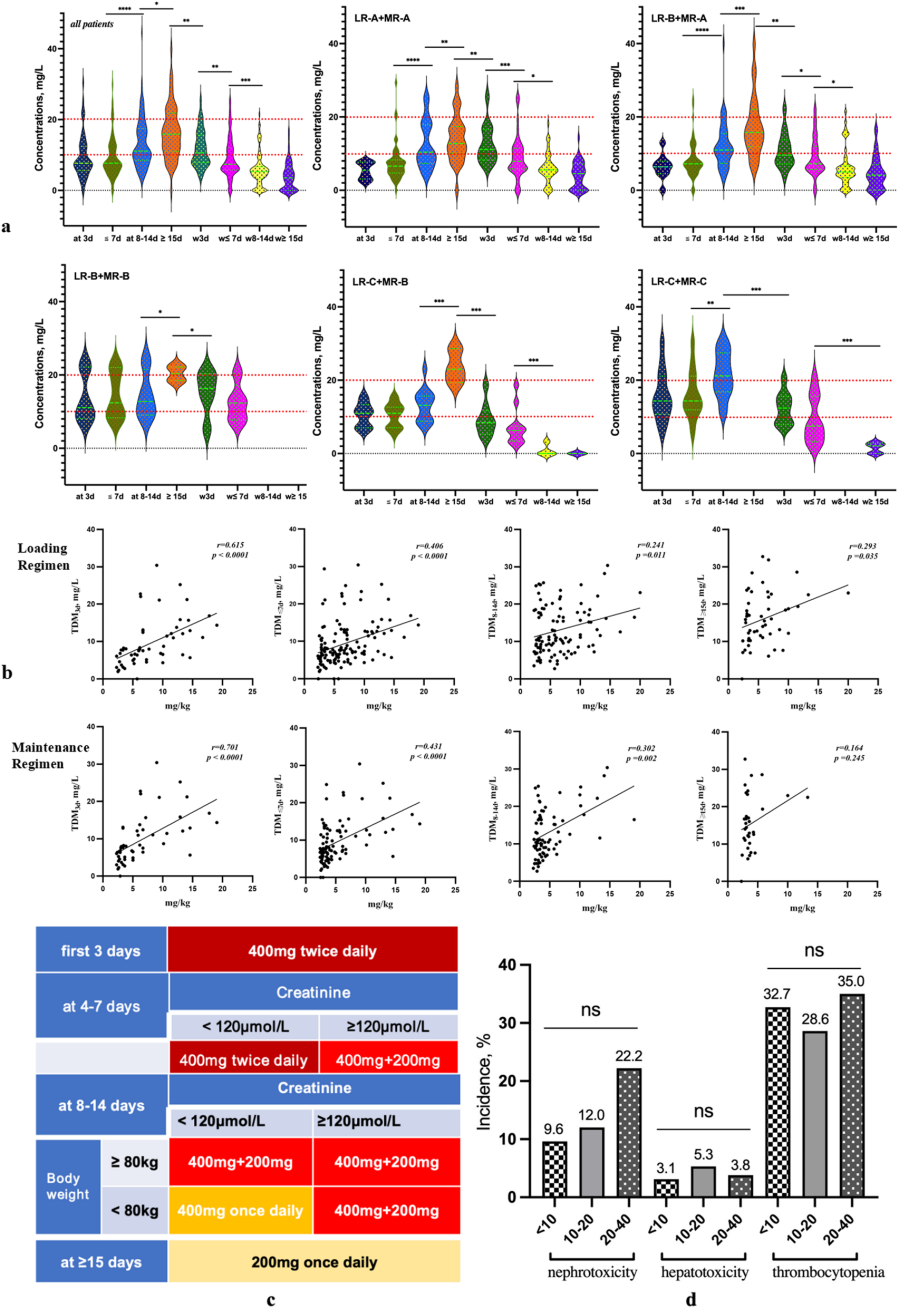
**a** Dynamic monitoring of teicoplanin concentrations. **b** Linear correlations between teicoplanin concentrations and the loading dose. TDM_3d_ had a significant linear correlation with the maintenance dose, TDM_≤7d_ had a moderate linear correlation with the maintenance dose, and TDM_8-14d_ had a slight linear correlation with the maintenance dose. **c** Recommended dose regimens based on the results of this study. **d** Incidence of teicoplanin related toxicities. There were no differences in the incidence of nephrotoxicity, hepatotoxicity, and thrombocytopenia among the C_min_ < 10 mg/L, C_min_ = 10–20 mg/L, and C_min_ = 20–40 mg/L groups

TDM_3d_ was 7.7 mg/L [5.6, 12.4], TDM_≤7d_ was 7.6 mg/L [5.6, 12.2], TDM_8-14d_ was 11.1 mg/L [8.5, 17.6], and TDM_≥15d_ was 15.8 mg/L [11.0, 21.8], with 42 (66.7%), 93 (66.4%), 44 (36.4%), and 10 patients (17.0%) having suboptimal concentrations, respectively (Table 2, Fig. 2a). There was no difference between TDM_3d_ and TDM_≤7d_; TDM_8-14d_ was significantly higher than TDM_3d_ and TDM_≤7d_, and TDM_≥15d_ was significantly higher than TDM_8-14d_.

**Table 2:** Dynamic monitoring of teicoplanin concentrations with different dose regimens

TDM, mg/L	All Patirents (*N* = 204)	Dose Regimens				
		LR-A + MR-A	LR-B + MR-A	LR-B + MR-B	LR-C + MR-B	LR-C + MR-C
At 3d	7.7[5.6,12.4]	5.4[3.0,7.7]	6.3[4.4,7.9]	11.1[8.1,22.2]	11.0[7.0,13.3]	14.4[10.7,21.1]
< 10	42(66.7)	16(100)	17(89.5)	3(50.0)	3(37.5)	3(21.4)
10–20	15(23.8)	0	2(10.5)	1(16.7)	5(62.5)	7(50.0)
> 20	6(9.5)	0	0	2(33.3)	0	4(28.6)
≤ 7d	7.6[5.6,12.2]	6.4[4.7,8.2]	7.2[5.5,10.0]	12.42[8.3,22.0]	11.0[7.0,12.6]	14.4[12.0,21.2]
< 10	93(66.4)	40(81.7)	41(75.9)	3(42.8)	7(43.8)	2(14.3)
10–20	35(25.0)	6(12.2)	10(18.5)	2(28.6)	9(56.2)	8(57.1)
> 20	12(8.6)	3(6.1)	3(5.6)	2(28.6)	0	4(28.6)
At 8-14d	11.1[8.5,17.6]	10.4[7.3,17.8]	11.0[7.3,15.4]	12.8[9.0,21.5]	14.16[8.9,16.4]	21.2[16.8,27.4]
< 10	44(36.4)	22(45.8)	16(34.0)	1(25.0)	5(35.7)	0
10–20	58(47.9)	20(41.7)	25(53.2)	2(50.0)	8(57.2)	3(37.5)
> 20	19(15.7)	6(12.5)	6(12.8)	1(25.0)	1(7.1)	5(62.5)
≥ 15d	15.8[11.0,21.8]	12.7[10.1,17.4]	15.7[11.9,22.0]	20.3[13.8,20.4]	23.0[19.4,28.6]	-
< 10	10(17.0)	6(22.2)	4(15.4)	0	0	
10–20	35(59.3)	16(59.3)	15(57.7)	1(50.0)	1(33.3)	
> 20	14(23.7)	5(18.5)	7(26.9)	1(50.0)	2(66.7)	
Withdraw 3d	10.6[7.9,15.6]	11.1[8.2,16.5]	9.6[7.2,13.8]	16.3[9.4,20.2]	8.4[5.3,11.7]	12.4[7.9,15.6]
< 10	23(46.9)	5(38.5)	7(50.0)	1(20)	6(75.0)	4(44.4)
10–20	22(44.9)	7(53.8)	6(42.9)	3(60)	2(25.0)	4(44.4)
> 20	4(8.2)	1(7.7)	1(7.1)	1(20)	0	1(11.2)
Withdraw ≤ 7d	7.4[5.7,12.0]	7.9[5.8,11.4]	7.2[5.6,12.0]	12.3[7.7,17.0]	6.3[3.4,9.4]	7.5[3.2,15.6]
< 10	78(66.1)	30(65.2)	31(66.0)	2(40)	9(81.8)	6(66.7)
10–20	33(28.0)	13(28.3)	14(29.8)	2(40)	2(18.2)	2(22.2)
> 20	7(5.9)	3(6.5)	2(4.2)	1(20)	0	1(11.1)
Withdraw 8-14d	5.3[3.3,7.0]	5.5[3.8,7.6]	5.0[3.4,6.6]	-	0[0,2.5]	-
< 10	87(84.5)	42(84.0)	40(83.3)		4(100)	
10–20	15(14.5)	8(16.0)	7(14.6)		0	
> 20	1(1.0)	0	1(2.1)		0	
Withdraw ≥ 15d	3.5[0,6.4]	4.54[0,7.3]	4.1[0,7.0]	-	0[0,0]	2.1[0,2.8]
< 10	86(94.5)	41(97.6)	34(89.5)		5(100)	5(100)
10–20	5(5.5)	1(2.4)	4(10.5)		0	0
> 20	0	0	0		0	0

*TDM* therapeutic drug monitoring, *LR* loading regimen, *MR* maintenance regimen

#### After withdrawal

TDM_w3d_ was 10.6 mg/L [7.9, 15.6], with 22 patients (44.9%) having concentrations of 10–20 mg/L; four (8.2%) had concentrations exceeding 20 mg/L. TDM_w≤7d_ was 7.4 mg/L [5.7, 12.0], with 33 patients (28.0%) having concentrations of 10–20 mg/L, and seven (5.9%) with concentrations exceeding 20 mg/L. TDM_w8-14d_ was 5.3 mg/L [3.3, 7.0]; 15 patients (14.6%) had concentrations of 10–20 mg/L, and one (1.0%) had a concentration exceeding 20 mg/L. TDM_w≥15d_ was 3.5 mg/L [0, 6.4], with five patients (5.5%) with concentrations of 10–20 mg/L (Table 2, Fig. 2a). TDM_w≤7d_ was significantly lower than TDM_w3d_, TDM_w8-14d_ was significantly lower than TDM_w≤7d_, and no difference was seen between TDM_w8-14d_ and TDM_w≥15d_ (Table 2, Fig. 2a).

### Dynamic monitoring of teicoplanin concentrations with different dose regimens

In the LR-A + MR-A regimen, the target concentration achievement rate for TDM_3d_ (≥ 10 mg/L) was 0%, compared with 18.3% for TDM_≤7d_, 54.2% for TDM_8-14d_, and 77.8% for TDM_≥15d_. In the LR-B + MR-A regimen, the rate for TDM_3d_ was 10.5%, compared with 24.1% for TDM_≤7d_, 66.0% for TDM_8-14d_, and 84.6% for TDM_≥15d_. For the LR-B + MR-B regimen, the rate for TDM_3d_ was 50.0% compared with 57.2% for TDM_≤7d_, 75.0% for TDM_8-14d_, and 100% for TDM_≥15d_. The LR-C + MR-B regimen had a rate for TDM_3d_ of 62.5%, compared with 56.2% for TDM_≤7d_, 64.3.0% for TDM_8-14d_, and 100% for TDM_≥15d_. In the LR-C + MR-C regimen, the achievement rate for TDM_3d_ was 78.6%, compared with 85.7% for TDM_≤7d_ and 100% for TDM_8-14d_ (Table 2, Fig. 2a). From LR-A + MR-A to LR-C + MR-C, the C_min_s during teicoplanin treatment and the target concentration achievement rates both gradually increased (Table 2, Fig. 2a).

### Linear relationship between dose regimens and concentrations

TDM_3d_ had a very linear significant correlation with the loading dose (*r* = 0.615, *P* < 0.0001; Fig. 2b), while TDM_≤7d_ had a moderately linear significant correlation with the loading dose (*r* = 0.406, *P* < 0.0001; Fig. 2b); these trends were the same with the maintenance dose (*r* = 0.701, *P* < 0.0001 and *r* = 0.431, *P* < 0.0001, respectively; Fig. 2b). TDM_8-14d_ had a slight linear correlation with the maintenance dose (*r* = 0.302, *P* = 0.002, Fig. 2b).

### Factors associated with suboptimal C_min_ and slow elimination

#### Suboptimal concentrations

For TDM_3d_, the maintenance dose (400 vs. 200 mg once daily: odds ratio [OR] = 0.014, 95% confidence interval [CI] = 0.001–0.222, *P* = 0.003; 400 mg twice daily vs. 200 mg once daily: OR = 0.003, 95% CI = 0.0001–0.079, *P* < 0.0001) was independently associated with suboptimal concentrations in multivariate analysis (Table 3). For TDM_≤7d_, the maintenance dose (400 vs. 200 mg once daily: OR = 0.095, 95% CI = 0.013–0.690, *P* = 0.020; 400 mg twice daily vs. 200 mg once daily: OR = 0.015, 95% CI = 0.001–0.250, *P* = 0.003) and creatinine < 120 μmol/L (OR = 7.361, 95% CI = 2.081–26.035, *P* = 0.002) were independently associated with suboptimal concentrations in multivariate analysis (Table 3). For TDM_8-14d_, body weight ≥ 80 kg (OR = 3.417, 95% CI = 1.135–10.280, *P* = 0.029) and creatinine < 120 μmol/L (OR = 4.619, 95% CI = 1.627–13.114, *P* = 0.004) were independently associated with suboptimal concentrations in multivariate analysis (Table 3).

**Table 3:** Factors associated with suboptimal trough concentrations and slow elimination in older adult patients during the use of teicoplanin

Variable	Univariate analysis		Multivariate analysis			
	Unstandardized β coefficient (95% CI)	*P*	Unstandardized β coefficient (95% CI)			*P*
Factors with suboptimal trough concentrations during treatment						
At 3 days (*n* = 63)						
Age, years	1.069(1.025–1.116)	0.002	0.947(0.876–1.023)			0.164
Gender, male						
Weight, kg	1.025(0.979–1.073)	0.284				
Body mass index, kg/m^2^	1.028(0.892–1.185)	0.700				
Loading Regimen (vs 200mg 1/ day)	0.171(0.035–0.834)	0.026	1.408(0.108–18.420)			0.794
400mg 1/ day or 2/ day						
Loading Regimen, mg/kg	0.656(0.532–0.810)	< 0.0001				
Maintenance Regimen (vs 200mg 1/ day)		< 0.0001				0.002
400mg 1/ day	0.045(0.008–0.269)	0.001	0.014(0.001–0.222)			0.003
400mg 2/ day	0.017(0.002–0.112)	< 0.0001	0.003(0.0001–0.079)			< 0.0001
Maintenance Regimen, mg/kg	0.655(0.520–0.826)	< 0.0001				
Duration, days	1.086(0.979–1.205)	0.120				
Laboratory findings at baseline						
Albumin, g/L	1.039(0.988–1.092)	0.134				
Creatinine < 120μmol/L	1.000(0.168–5.956)	1.000				
eGFR ≥ 60ml/min/1.73m^2^	2.273(0.739–6.992)	0.152				
Bilirubin, μmol/L	1.004(-.985–1.024)	0.659				
ALT, U/L	0.983(0.962–1.004)	0.120				
≤ 7 days (*n* = 140)						
Age, years	1.047(1.017–1.077)	0.002	0.943(0.886–1.004)		0.067	
Gender, male	2.537(0.801–8.037)	0.113				
Weight, kg	1.010(0.981–1.040)	0.511				
Body mass index, kg/m^2^	0.959(0.873–1.054)	0.387				
Loading Regimen (vs 200mg 1/ day)		< 0.0001			0.601	
400mg 1/day	0.582(0.233–1.253)	0.246	0.595(0.214–1.658)		0.312	
400mg 2/day	0.096(0.033–0.280)	< 0.0001	0.494(0.057–4.287)		0.494	
Loading Regimen, mg/kg	0.772(0.687–0.869)	< 0.0001				
Maintenance Regimen(vs 200mg 1/ day)		< 0.0001			0.013	
400mg 1/ day	0.209(0.081–0.540)	0.001	0.095(0.013–0.690)		0.020	
400mg 2/ day	0.045(0.009–0.217)	< 0.0001	0.015(0.001–0.250)		0.003	
Maintenance Regimen, mg/kg	0.701(0.592–0.850)	< 0.0001				
Duration, days	1.003(0.958–1.049)	0.904				
Laboratory findings at baseline						
Albumin, g/L	1.011(0.985–1.038)	0.409				
Creatinine < 120μmol/L	4.757(1.521–14.875)	0.007	7.361(2.081–26.035)		0.002	
eGFR ≥ 60ml/min/1.73m^2^	1.633(0.817–3.385)	0.161				
Bilirubin, μmol/L	0.998(0.987–1.010)	0.767				
ALT, U/L	0.998(0.994–1.002)	0.270				
At 8–14 days (*n* = 121)						
Age, years	1.027(0.991–1.064)	0.143	1.038(0.977–1.103)		0.232	
Gender, male	-	0.999				
Weight, kg	1.029(0.996–1.064)	0.087				
Weight ≥ 80 kg		0.094	3.417(1.135–10.280)		0.029	
Body mass index, kg/m^2^	1.071(0.965–1.189)	0.197				
Loading Regimen (vs 200mg 1/ day)	0.510(0.239–1.086)	0.081	0.434(0.165–1.136)		0.189	
400mg 1/ day or 2/ day						
Loading Regimen, mg/kg	0.849(0.749–0.962)	0.010				
Maintenance Regimen (vs 200mg 1/ day)	0.450(0.165–1.224)	0.118	0.692(0.143–3.347)		0.647	
400mg 1/ day or 2/ day						
Maintenance Regimen, mg/kg	0.689(0.512–0.926)	0.013				
Duration, days	1.001(0.956–1.047)	0.975				
Laboratory findings at baseline						
Albumin, g/L	1.005(0.981–1.030)	0.673				
Creatinine < 120μmol/L	2.350(0.989–5.583)	0.053	4.619(1.627–13.114)		0.004	
eGFR ≥ 60ml/min/1.73m^2^	0.901(0.429–1.894)	0.783				
Bilirubin, μmol/L	1.005(0.989–1.021)	0.549				
ALT, U/L	0.980(0.953–1.006)	0.133				
Factors with slow elimination after drug withdrawal						
Withdraw ≤ 7 days (*n* = 118)						
Age, years	1.021(0.987–1.056)	0.228				
Gender, male	0.227(0.053–0.960)	0.044		0.560(0.080–3.909)		0.559
Weight, kg	0.988(0.957–1.020)	0.460				
Body mass index, kg/m^2^	0.971(0.879–1.073)	0.567				
Loading Regimen (vs 200mg 1/ day)	0.846(0.389–1.836)	0.672				
400mg 1/ day or 2/ day						
Loading Regimen, mg/kg						
Maintenance Regimen (vs 200mg 1/ day)	0.969(0.375–2.505)	0.948				
400mg 1/ day or 2/ day						
Maintenance Regimen,mg/kg						
TDM_a_, mg/L	1.283(1.135–1.450)	< 0.0001				
TDM_a_ ≥ 15mg/L	9.383(3.215–27.379)	< 0.0001	10.374(3.338–32.242)			< 0.0001
Duration, days	1.054(1.002–1.109)	0.041	1.034(0.962–1.111)			0.366
Laboratory findings at baseline						
Albumin, g/L	0.994(0.967–1.022)	0.680				
Creatinine ≥ 120μmol/L	2.520(1.074–5.911)	0.034				
eGFR < 60ml/min/1.73m^2^	2.400(1.100–5.235)	0.028	1.992(0.627–6.333)			0.243
Bilirubin, μmol/L	1.005(0.996–1.015)	0.266				
ALT, U/L	1.000(0.995–1.005)	0.978				
Withdraw 8–14 days (*n* = 103)						
Age, years	1.040(0.951–1.138)	0.389				
Gender, male	0.353(0.030–4.141)	0.407				
Weight, kg	0.969(0.921–1.019)	0.215				
Body mass index, kg/m^2^	0.916(0.783–1.072)	0.275				
TDM_a_, mg/L	1.270(1.121–1.440)	< 0.0001				
TDM_a_ ≥ 15mg/L	24.000(4.673–123.263)	< 0.0001	47.106(4.130–537.231)			0.002
Loading Regimen (vs 200mg 1/ day)	0.977(0.336–2.839)	0.966				
400mg 1/ day or 2/ day						
Loading Regimen, mg/kg						
Maintenance Regimen (vs 200mg 1/ day)	-	0.999				
400mg 1/ day or 2/ day						
Maintenance Regimen, mg/kg						
Duration ≥ 15 days	4.909(1.462–16.489)	0.010	11.082(0.967–126.930)			0.053
Laboratory findings at baseline						
Albumin < 38g/L	0.948(0.896–1.003)	0.065	12.947(0.860–226.185)			0.064
Creatinine ≥ 120μmol/L	2.662(0.844–8.394)	0.095				
eGFR < 60ml/min/1.73m^2^	7.091(1.879–26.757)	0.004	23.657(1.584–353.231)			0.022
Bilirubin, μmol/L	1.003(0.985–1.021)	0.769				
ALT, U/L	1.000(0.992–1.008)	0.976				

*TDM* therapeutic drug monitoring, *COPD* chronic obstructive pulmonary disease, *CKD* chronic kidney disease, *ALT* Alanine aminotransferase, *eGFR* estimated glomerular filtration rate (CKD-EPI), *SOFA* Sequential organ failure assessment

#### Slow elimination

For TDM_w≤7d_, TDM_a_ ≥ 15 mg/L (OR = 10.374, 95% CI = 3.338–32.242, *P* < 0.0001) was associated with slow elimination in multivariate analysis (Table 3), while for TDM_w8-14d_, TDM_a_ ≥ 15 mg/L (OR = 47.106, 95% CI = 4.130–537.231, *P* = 0.002) and eGFR < 60 mL/min/1.73 m^2^ (OR = 23.657, 95% CI = 1.584–353.231, *P* = 0.022) were associated with slow elimination.

### Optimal regimen

A dose of 400 mg at 12-h intervals was determined for the first 3 days (six doses). On days 4–7, the recommended dose was changed to 400 mg at 12-h intervals when creatinine is < 120 μmol/L, or alternating doses of 400 and 200 mg at 12-h intervals (400 mg + 200 mg daily) when creatinine is ≥ 120 μmol/L. On days 8–14, when creatinine is < 120 μmol/L or body weight is ≥ 80 kg, the recommended dose was 400 mg + 200 mg daily; otherwise, it was 400 mg once daily. On and after day 15, the dose recommendation was 200 mg once daily (Fig. 2c).

### Teicoplanin-related toxicities

The incidence of acute kidney injury (AKI) was 12.5% (15/120); it was 9.6% (5/52) when TDM_a_ < 10 mg/L, 12.0% (6/50) when TDM_a_ = 10–20 mg/L, and 22.2% (4/18) when TDM_a_ = 20–40 mg/L (*P* = 0.424, Fig. 2d). The incidence of hepatotoxicity was 4.1% (6/148); it was 3.1% (2/65) when TDM_a_ < 10 mg/L, 5.3% (3/57) when TDM_a_ = 10–20 mg/L, and 3.8% (1/26) when TDM_a_ = 20–40 mg/L (*P* = 0.862, Fig. 2d). The incidence of thrombocytopenia was 31.5% (35/111); it was 32.7% (16/49) when TDM_a_ < 10 mg/L, 28.6% (12/42) when TDM_a_ = 10–20 mg/L, and 35.0% (7/20) when TDM_a_ = 20–40 mg/L (*P* = 0.893, Fig. 2d).

## Discussion

Our study provides a critical insight into the teicoplanin dosing regimens for older adults in Beijing, demonstrating prevalent underdosing; over 80% of patients received reduced doses, with 40% of patients failing to receive loading doses. This dosing conservatism significantly contributed to the suboptimal therapeutic levels observed in 66.4% of patients within the first week of treatment, with a gradual increase in concentration over time indicating drug accumulation. Given the varied risk factors for suboptimal concentrations in different treatment stages, a one-size-fits-all regimen was ineffective. In addition, we found that the rates of nephrotoxicity, hepatotoxicity, and thrombocytopenia did not increase with concentration when C_min_ ≤ 40 mg/L.

There are few studies on the optimal dose regimen and target concentration of teicoplanin in older adults. Wang et al. [9] examined 18 cases of patients aged ≥ 65 years and found the half-life of teicoplanin was 71–80 h. Rosina et al. [26] studied the pharmacokinetics of teicoplanin in 12 patients aged ≥ 65 years old and found that the average elimination half-life was 107 h. Kang et al. [27] examined 15 cases of critically ill patients ≥ 60 years of age receiving teicoplanin (a loading dose of 6 mg/kg administered every 12 h for the initial three doses, followed by a 6 mg/kg daily maintenance dose) and found that the steady C_min_ was 8.7 [7.2–9.5] mg/L. They recommended that high-dose regimens should be considered as empiric therapy for critically ill older adult patients; however, the number of cases included was small, and the dose regimens and concentrations of teicoplanin used were not well described, meaning that further research is necessary.

Our results show that 83.3% of patients received a reduced dose of teicoplanin, and C_min_ gradually increased with the duration of treatment. Severe underexposure occurred within 14 days of reduced-dose teicoplanin treatment in older adult patients. Interestingly, C_min_ increased significantly after 14 days of administration, with more than 80% of patients achieving therapeutic concentrations, suggesting that a minimum maintenance dose of 200 mg once daily is appropriate after 14 days. The half-life of teicoplanin ranged from 71–163 h, and the time to reach steady state was 4–5 half-lives if the drug was given at regular intervals [28]. Steady-state teicoplanin concentrations were obtained in 93% of patients after 14 days of repeated administration [28]. Byrne et al. [29] also reported that teicoplanin C_min_ was positively associated with the day of therapy, indicating significant drug accumulation.

In this study, the C_min_ at 3 days of treatment was significantly higher in the high-loading dose regimen (400 mg twice daily), suggesting that a high loading dose was mandatory to achieve optimal drug concentration [3, 10, 12]. In addition, we found that the C_min_ at 3 days of treatment was not correlated with renal function, consistent with the recommendation in the instructions and guidelines stating that the loading dose in the first 3 days should not be adjusted according to renal function; this is also in line with teicoplanin pharmacokinetics. The C_min_ was independently associated with serum creatinine within the first 7 days of treatment as well as with body weight and serum creatinine levels at 8–14 days of treatment, suggesting that the dosing regimen could be adjusted according to body weight and serum creatinine levels. We did not find a correlation between C_min_ and eGFR; however, the observed correlation between C_min_ and serum creatinine levels contradicted previous findings [12, 30–32]. We did not believe that serum creatinine accurately reflected renal function, but it could be representative of the drug concentration. Our results were consistent with previous studies [12, 30, 33, 34]. The cumulative urinary excretion of teicoplanin is decreased and the half-life is enhanced by renal impairment [33]. Wang et al. [12] suggested that teicoplanin dose regimens in intensive care unit patients should be stratified by renal function. Another study by Byrne et al. [8] recommended individualized dose regimens based on body weight and creatinine clearance to guarantee optimal teicoplanin concentrations. It has been demonstrated that hypoalbuminemia can influence teicoplanin C_min_ [30, 31]. However, we did not find a relationship between teicoplanin concentrations and albumin, which may be attributed to the generally low albumin seen in older adults. Considering drug accumulation and the risk factors for suboptimal concentrations at different stages, we recommended a dose of 400 mg every 12 h for the first 3 days (six doses); on days 4–7, the recommended dose is 400 mg every 12 h when creatinine is < 120 μmol/L, otherwise, 400 mg + 200 mg daily should be used. On days 8–14, when creatinine is < 120 μmol/L or body weight is ≥ 80 kg, the regimen should be 400 mg + 200 mg daily; otherwise, 400 mg should be given once daily. On and after day 15, the dose should be 200 mg daily. Considering variabilities in tecoplanin pharmacokinetics in older adults, TDM is still recommended.

We dynamically monitored teicoplanin concentrations after drug withdrawal, which has also been done in a few previous studies. Nearly 34% of patients displayed therapeutic concentrations (7.42 mg/L [5.76, 12.03]) within the first 7 days after withdrawal, and teicoplanin remained detectable in two-thirds of patients ≥ 15 days after withdrawal. We also found that slow elimination was associated with TDM_a_ and eGFR. Wang et al. [9] monitored teicoplanin concentrations in 18 older adult patients after drug withdrawal and found that the concentration exceeded 10 mg/L 9 days after treatment cessation. This slow elimination emphasizes the importance of continuous monitoring for potential toxicity, suggesting that vigilance should extend into the post-treatment period, especially considering teicoplanin’s high binding affinity and extended half-life [28].

The incidence of adverse events such as nephrotoxicity, hepatotoxicity, and thrombocytopenia did not significantly increase with higher trough concentrations of teicoplanin (C_min_ ≤ 40 mg/L), suggesting that its safety profile may be more favorable than anticipated at higher doses. This was consistent with research by Ueda [17] and Seki [3] and challenges the prevailing caution against dose escalation because of toxicity fears, advocating for a balanced approach that considers both efficacy and safety.

This multicenter prospective study included a larger number of patients over 90 years of age than any other study examining teicoplanin concentrations. Despite this, some limitations must be acknowledged. First, the number of participants was relatively small, which could bias results and cause misinterpretations. Second, because older adult patients often have multiple pathogenic microbial infections (such as those caused by fungi or Gram-negative bacteria), we could not evaluate the relationship between teicoplanin concentration and treatment efficacy.

## Conclusions

Our findings highlight a significant issue with the current teicoplanin dosing regimens for older adults in China, revealing prevalent underdosing that may compromise therapeutic efficacy. This study underscores the necessity of personalized dosing strategies tailored to individual patient characteristics, such as renal function and body weight, to achieve optimal therapeutic efficacy.

Our data suggest that higher teicoplanin concentrations, achieved through adjusted dosing, do not significantly increase the risk of adverse events within the observed range; this challenges the cautious stance against higher dosing because of toxicity fears, supporting the safety of such an approach.

## Data Availability

To protect study participant privacy, our data cannot be shared openly. But the data are available from the corresponding author on reasonable request.

## Abbreviations

C_min_: Trough concentration
MRSA: Methicillin-resistant* S. aureus*
eGFR: Estimated glomerular filtration rate
TDM: Therapeutic drug monitoring
LRs: Loading regimens
MRs: Maintenance regimens (MRs)
OR: Odds ratio
CI: Confidence interval
AKI: Acute kidney injury
